# An Intracellular Threonine of Amyloid-β Precursor Protein Mediates Synaptic Plasticity Deficits and Memory Loss

**DOI:** 10.1371/journal.pone.0057120

**Published:** 2013-02-22

**Authors:** Franco Lombino, Fabrizio Biundo, Robert Tamayev, Ottavio Arancio, Luciano D’Adamio

**Affiliations:** 1 Department of Microbiology & Immunology, Albert Einstein College of Medicine, Bronx, New York, United States of America; 2 Department of Pathology & Cell Biology, Taub Institute for Research on Alzheimer’s Disease and the Aging Brain, Columbia University, New York, New York, United States of America; Cleveland Clnic Foundation, United States of America

## Abstract

Mutations in *Amyloid-*ß *Precursor Protein (APP)* and *BRI2/ITM2b* genes cause Familial Alzheimer and Danish Dementias (FAD/FDD), respectively. APP processing by BACE1, which is inhibited by BRI2, yields sAPPß and ß-CTF. ß-CTF is cleaved by gamma-secretase to produce Aß. A knock-in mouse model of FDD, called FDD_KI_, shows deficits in memory and synaptic plasticity, which can be attributed to sAPPß/ß-CTF but not Aß. We have investigated further the pathogenic function of ß-CTF focusing on Thr^668^ of ß-CTF because phosphorylation of Thr^668^ is increased in AD cases. We created a knock-in mouse bearing a Thr^668^Ala mutation (*APP^TA^* mice) that prevents phosphorylation at this site. This mutation prevents the development of memory and synaptic plasticity deficits in FDD_KI_ mice. These data are consistent with a role for the carboxyl-terminal APP domain in the pathogenesis of dementia and suggest that averting the noxious role of Thr^668^ is a viable therapeutic strategy for human dementias.

## Introduction

Familial dementias are caused by mutations in *APP* [1] and genes that regulate APP processing. These include the *PSEN1/2* genes, which code for the catalytic component of the gamma-secretase, and the *BRI2/ITM2b* gene, whose protein product BRI2 binds APP and inhibits APP processing [1]–[8]. Cases caused by *APP/PSEN* mutations are classified as FAD and those caused by mutations in *BRI2/ITM2b* as FDD or Familial British dementia (FBD). The prevailing pathogenic model for these dementias posits that amyloid peptides trigger dementia. In AD, the amyloid peptide Aß is a part of APP; in FDD and FBD, the amyloidogenic peptides, called ADan and ABri respectively, are generated from the mutant BRI2 proteins [2], [8]. FDD patients present mixed amyloid plaques containing both Aβ and ADan. However, recent data suggest that these dementias share pathogenic mechanisms involving synaptic-toxic APP metabolites distinct from Aβ [9], [10].

In FDD, a 10-nucleotide duplication in the *BRI2/ITM2B* gene leads to the synthesis of a longer BRI2 protein [8]. In normal individuals, BRI2 is synthesized as an immature type-II membrane protein (imBRI2) that is cleaved at the C-terminus into mature BRI2 and a 23aa soluble C-terminal fragment [11]. In FDD patients, cleavage of the BRI2 mutant protein leads to the release of the longer ADan peptide [8]. To model FDD we generated FDD_KI_ mice that like FDD patients [8], carry one wild type *Bri2/Itm2b* allele and the other one has the Danish mutation [12]. FDD_KI_ mice develop synaptic and memory deficits due to loss of Bri2 protein, but do not develop amyloidosis [13]. BRI2 binds to APP and inhibits cleavage of APP by secretases [4]–[7]. Owing to the loss of BRI2, processing of APP is increased in FDD [14], [15]. Memory and synaptic deficits of FDDKI mice require APP [14], and are mediated by sAPPß and/or ß-CTF produced during synaptic plasticity and memory acquisition. Inhibition of γ-secretase, the enzyme that processes β-CTF to yield Aß, worsens memory deficits and is associated with an accumulation of ß-CTF [10], [16], [17]. In addition, caspase-9 in activated in FDD_KI_ mice and caspase-9 activity mediates memory/synaptic plasticity deficits [18]. Overall, these results suggest that ß-CTF, rather than Aß, is a major toxic species causing dementia. Here, we have investigated further the pathogenic role of the carboxyl-terminal region of APP and especially the role of residue Thr^668^.

## Results

### Thr^668^ of APP Mediates Object Recognition Deficits found in FDD_KI_ Mice

**Figure 1:**
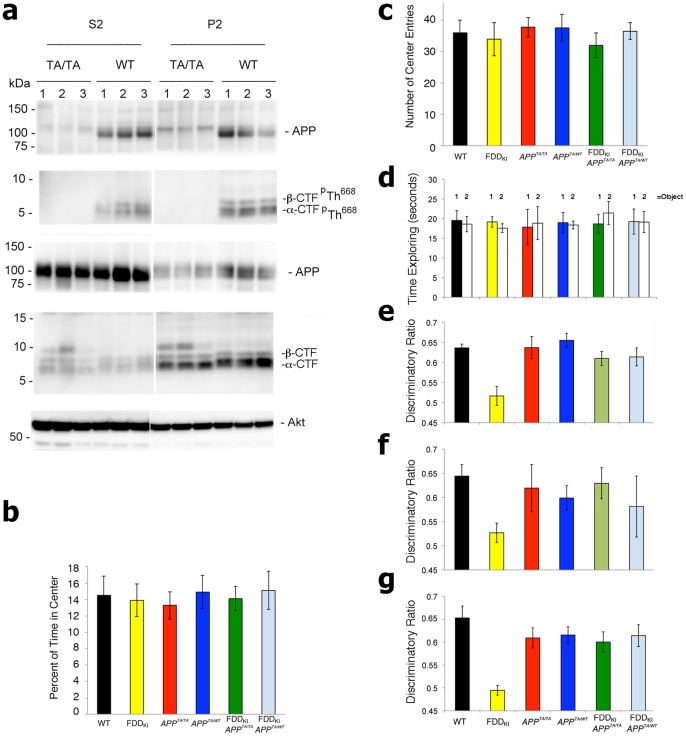
A Thr^668^Ala mutation on APP prevents the object recognition memory deficit of FDD_KI_ mice. (**a**) Western blot analysis of hippocampal synaptosomal preparations shown that the Thr to Ala mutation abolishes phosphorylation of Thr^668^ (APP^p^Thr^668^). Interestingly, only the mature form of APP (mAPP) and not the immature (imAPP), is found phosphorylated on this Thr in hippocampal synaptic fractions of WT mice. (**b** and **c**) Open field is a sensorimotor test for habituation, exploratory, emotional behavior, and anxiety-like behavior, in novel environments. The percent of time in the center (b) and the number of entries into the center (c) are indicators of anxiety levels. The more the mouse enters the center and explores it, the lower the level of anxiety-like behavior. Since the FDD_KI_, FDD_KI_/*APP^TA/TA^*, FDD_KI_/*APP^TA/WT^*, *APP^TA/TA^*, *APP^TA/WT^* mice are similar to the WT animals there is no deficit or excess of anxiety. (**d**) All six genotypes (WT, FDD_KI_, FDD_KI_/*APP^TA/TA^*, FDD_KI_/*APP^TA/WT^*, *APP^TA/TA^*, *APP^TA/WT^*) mice spent similar amounts of time exploring the two identical objects on day 1. (**e**) FDD_KI_/APP^TA/TA^ and FDD_KI_/APP^TA/WT^ mice behaved similarly to WT mice and prevented the deficit in the NOR tests found in FDD_KI_ mice at 6 months of age (FDD_KI_ versus FDD_KI_/APP^TA/TA^ P = 0.011; FDD_KI_ versus FDD_KI_/APP^TA/WT^ P = 0.0083; FDD_KI_ versus WT P<0.001), (**f)** 9 months of age (FDD_KI_ versus FDD_KI_/APP^TA/TA^ P = 0.01; FDD_KI_ versus FDD_KI_/APP^TA/WT^ P = 0.347; FDD_KI_ versus WT P = 0.000995), and (**g**) 12 months of age (FDD_KI_ versus FDD_KI_/APP^TA/TA^ P = 0.0003; FDD_KI_ versus FDD_KI_/APP^TA/WT^ P = 0.0002; FDD_KI_ versus WT P<0.0001). Thus the APP^TA^ point mutation prevented the novel object recognition deficit of FDD_KI_ mice.

Recent findings suggest that products of BACE1-processing of APP (predominantly ß-CTF) trigger several pathological features related to human dementias both in a mouse model of FDD [10], [16] and human neurons derived from familial and sporadic AD [9]. Thus, we decided to probe in more details the pathogenic function of the carboxyl-terminal region of APP, focusing on the intracellular Thr^668^ residue (following the numbering of the APP^695^ isoform). The phosphorylation status of Thr^668^ either creates or destroys docking sites for intracellular proteins that interact with APP [19]–[22]. In addition, phosphorylation at Thr^668^ is increased in AD cases [23] suggesting potential pathogenic implications. We generated mice expressing APP with a Thr^668^Ala mutation, called *APP^TA^* [24]. Western blot analysis of hippocampal synaptosomes from either *APP^WT/WT^* or *APP^TA/TA^* mice shows that the Thr^668^Ala mutation abolishes phosphorylation at Thr^668^ (Figure 1a).

Thus, the *APP^TA^* mice are an ideal genetic tool to study the role of Thr^668^ and its phosphorylation in the pathogenesis of dementia. To this end, we utilized FDD_KI_ mice, which develop severe aging-dependent memory and synaptic plasticity deficits that first become measurable at ∼5 months of age [13]. Most importantly, these deficits are prevented when FDD_KI_ mice lack one allele of *APP*, reducing the APP protein load [14], and require production of APP ß-CTF [10], [16]. Thus, since memory and synaptic deficits of FDD_KI_ mice are dependent on endogenous APP, we can test the pathogenic role of Thr^668^ by introducing this APP mutation on the FDD_KI_ background.

By crossing FDD_KI_/*APP^TA/WT^* to *APP^TA/WT^* mice we generated littermates of the following 6 genotypes: WT, FDD_KI_, FDD_KI_/*APP^TA/TA^*, FDD_KI_/*APP^TA/WT^*, *APP^TA/TA^* and *APP^TA/WT^*. To test memory, six-month-old mice were subjected to the novel object recognition (NOR) task, which is a non-aversive task that relies on the mouse’s natural exploratory behavior. Open field studies showed that FDD_KI_, FDD_KI_/*APP^TA/TA^*, FDD_KI_/*APP^TA/WT^*, *APP^TA/TA^* and *APP^TA/WT^* mice have no defects in habituation and locomotor behavior, sedation, risk assessment and anxiety-like behavior in novel environments (Figure 1b and c). During the training session, mice of all genotypes spent the same amount of time exploring the two identical objects during the training phase (Figure 1d). The following day, when a novel object was introduced, FDD_KI_ spent the same amount of time exploring the two objects as if they were both novel to them, while the WT, *APP^TA/TA^*, and *APP^TA/WT^* mice still spent more time exploring the novel object (Figure 1e). Notably, FDD_KI_/*APP^TA/TA^* and FDD_KI_/*APP^TA/WT^* mice behaved like the WT mice and explored preferentially the novel object (Figure 1e), demonstrating a prevention of the defect of the FDD_KI_ mice. We subjected the mice to the NOR task at 9 months, and also at 12 months to confirm that this is a true prevention of deficits and not a delay. We found similar data to the data at 6 months with the FDD_KI_ mice showing no preference between the two objects on the second day, while the FDD_KI_/*APP^TA/TA^*, FDD_KI_/*APP^TA/WT^*, *APP^TA/TA^*, *APP^TA/WT^* mice all behaved similar to the WT mice (Figure 1f and 1g). These data confirm that memory is impaired in FDD_KI_ mice upon aging in an ethologically relevant, non-aversive behavioral context; remarkably, development of this deficit is fully prevented by changing the Thr^668^ residue on the intracellular region of APP to an Alanine.

### Thr^668^ of APP Mediates Short-term Memory Deficits Found in FDD_KI_ Mice

**Figure 2:**
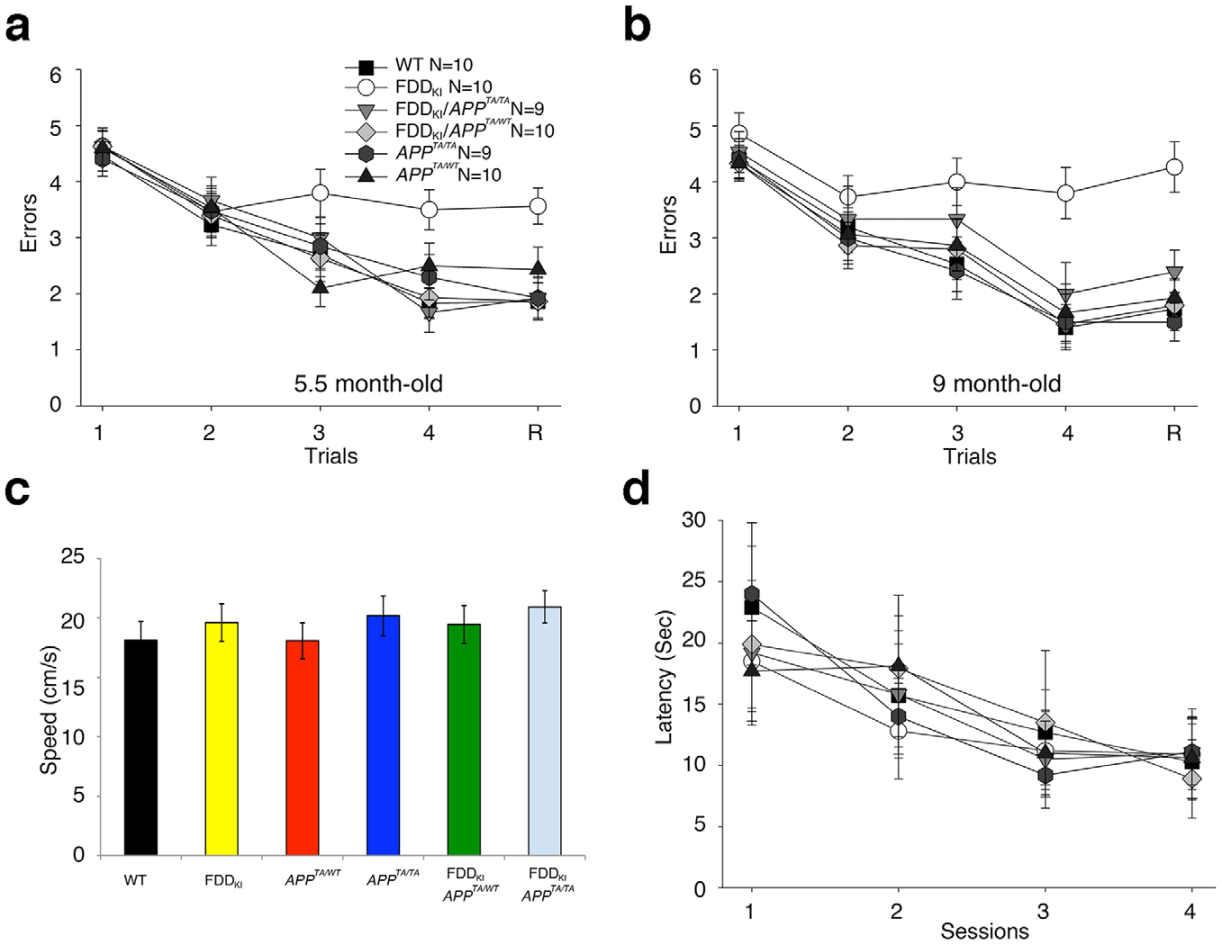
A Thr^668^Ala mutation on APP prevents the short-term memory deficit of FDD_KI_ mice. (**a** and **b**) In RAWM testing, FDD_KI_/*APP^TA/TA^*, FDD_KI_/*APP^TA/WT^*, *APP^TA/TA^*, *APP^TA/WT^* mice made the same number of errors as WT mice at both 5.5 months and 9 months of age. At 5.5 months of age, FDD_KI_ mice made significantly more errors at A4 (versus FDD_KI_/APP^TA/TA^ *P* = 0.0007; versus FDD_KI_/APP^TA/WT^ P = 0.0028; versus WT *P = *0.0005) and R (versus FDD_KI_/APP^TA/TA^ *P* = 0.0017; versus FDD_KI_/APP^TA/WT^ P = 0.0005; versus WT *P* = 0.0005) (**a**). Similar results are found at 9 months of age; FDD_KI_ mice made significantly more errors at A4 (versus FDD_KI_/APP^TA/TA^ *P* = 0.0004; versus FDD_KI_/APP^TA/WT^ P = 0.019; versus WT *P* = 0.0003) and R (versus FDD_KI_/APP^TA/TA^ *P* = 0.0006; versus FDD_KI_/APP^TA/WT^ P = 0.004; versus WT *P*<0.0001). Thus, the APP^TA/TA^ and APP^TA/WT^ point mutations prevent the development of working memory deficits in FDD_KI_ mice (**b**). (**c** and **d**) WT, FDD_KI_, FDD_KI_/*APP^TA/TA^*, FDD_KI_/*APP^TA/WT^*, *APP^TA/TA^* and *APP^TA/WT^* mice have similar speed (c) and need similar time (d) to reach a visible platform.

To further test memory, WT, FDD_KI_, FDD_KI_/APP^TA/TA^, FDD_KI_/APP^TA/WT^, APP^TA/TA^, APP^TA/WT^ mice were subjected at 5.5 months of age to the radial arm water maze (RAWM) task, a spatial working memory test that depends upon hippocampal function [25]. This task tests short-term memory, which is the memory affected in early stages of AD. The six genotypes were required to learn and memorize the location of a hidden platform in one of the arms of a maze with respect to spatial cues. WT, *APP^TA/TA^*, and *APP^TA/WT^* mice were able to acquire (A) and retain (R) memory of the task. FDD_KI_ mice showed severe abnormalities during both acquisition and retention of the task (Figure 2a), confirming that FDD_KI_ mice have severe impairment in short-term spatial memory for platform location during both acquisition and retention of the task. This defect was due to a deficit in memory *per se* and not to deficits in vision, motor coordination or motivation because testing with the visible platform showed no difference in the swimming speed and the time needed to find the platform between the FDD_KI_ and WT mice (Figure 2c and d). Both the FDD_KI_/*APP^TA/TA^* and the FDD_KI_/APP^TA/WT^ mice showed no defects in the memory test (Figure 2a), showing that mutating the intracellular APP residue Thr^668^ to an alanine prevented the RAWM deficit of FDD_KI_ mice, and confirming the data seen in NOR. To ensure that this was not simply a delay of the deficit, the mice were re-tested at 9 months in the RAWM task, and once again the FDD_KI_/*APP^TA/TA^* and the FDD_KI_/*APP^TA/WT^* mice did not show the deficit seen in the FDD_KI_ mice (Figure 2b).

### Thr^668^ of APP Mediates Synaptic Deficits Found in FDD_KI_ Mice

**Figure 3:**
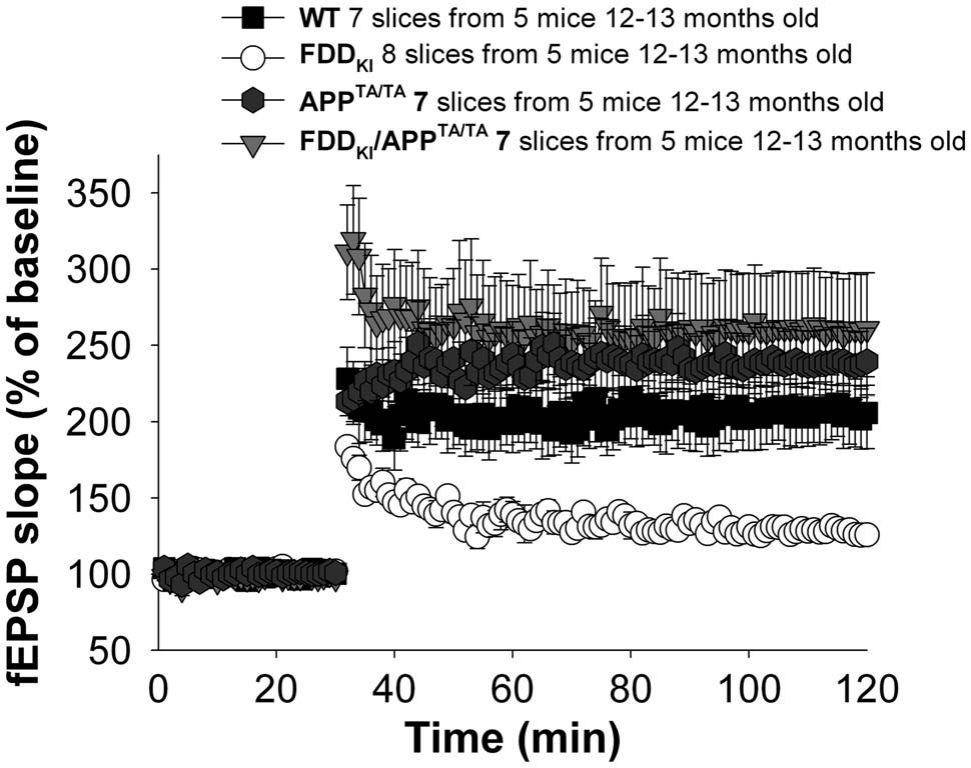
A Thr^668^Ala mutation on APP prevents the synaptic deficits of FDD_KI_ mice. Normal LTP in FDD_KI_/*APP^TA/TA^* and *APP^TA/TA^* compared with WT mice by two-way ANOVA (FDD_KI_/*APP^TA/TA^* versus WT mice: F(1,12) = 1.936; P = 0.187; *APP^TA/TA^* versus WT F(1,12) = 0.989; P = 0.338). Two-way ANOVA shows impaired LTP in FDD_KI_ mice when compared with WT (F(1,13) = 15.125; P = 0.002), to FDD_KI_/*APP^TA/TA^* (F(1,13) = 12.759; P = 0.004) or to *APP^TA/TA^* mice littermates (F(1,13) = 22.396; P<0.0001).

The FDD_KI_ mice have compromised long-term potentiation (LTP) [13], a long-lasting form of synaptic plasticity that is thought to be associated with learning and memory. Like for memory, the LTP deficit of FDD_KI_ mice are prevented when APP protein levels are halved [14], and by inhibiting processing of APP by BACE1 (also known as β-secretase) [10], [16]. Thus, we tested if this one amino acid change in APP could also prevent the synaptic plasticity defect found in the FDD_KI_ mice. To this end, we investigated synaptic transmission and plasticity using the Schaeffer collateral pathway in hippocampal slices from WT, *APP^TA/TA^*, FDD_KI_ and FDD_KI_/*APP^TA/TA^* mice. As expected, LTP was reduced in FDD_KI_ mice compared with WT littermates (Figure 3). Strikingly, the *APP^TA/TA^* point mutation prevented LTP impairments in FDD_KI_ mice (Figure 3). Taken together, these findings provide compelling genetic evidence that APP and BRI2 functionally interact, and that the synaptic and memory deficiencies due to loss of Bri2 function require the APP intracellular residue Thr^668^.

## Discussion

In this manuscript, we have pinpointed an intracellular residue of APP that is required for memory and synaptic plasticity deficits. FDD_KI_ mice allow for a genetic analysis of pathogenic pathways on a genetic background that is congruous to the human disease. We showed that haplodeficiency in APP prevented all FDD_KI_ mice’s deficits at all ages. Now we take this further by showing that mutation in just one residue of APP, the intracellular amino acid Thr^668^, can also prevent the memory and synaptic deficits.

We studied the functional relevance of Thr^668^ of APP because APP^p^Thr^668^ is enriched in AD patients [23], suggesting a pathogenic role for phosphorylation at this residue, and because it has profound effects on APP protein/protein interactions and APP biology. For example, Thr^668^ phosphorylation impairs APP/Fe65 interaction [20], [21] but promotes Pin1 binding [22]. In addition, this phosphorylation regulates trafficking of APP and APP derived metabolites [26]. Previous studies in mice suggested a protective role for phosphorylation of Thr^668^ in the pathogenesis of AD by showing that Pin1 decreases APP processing and Aß production by binding APP phosphorylated on Thr^668^ [27]. However, analysis of the *APP^TA^* mice has shown that preventing phosphorylation by mutating Thr^668^ into an Ala does not change Aß levels *in vivo* [28], [29].

If Aß were a major neuro-toxic peptide in dementia, FDD_KI_/*APP^TA/TA^* mice should either have deficits comparable to FDD_KI_ mice based on the evidence that the Thr^668^Ala mutation does not change Aß levels [28], [29], or should present with a worsened phenotype based on the hypothesis that binding of Pin1 to APP^p^Thr^668^ reduces Aß levels [27]. Instead, we have found that the Thr^668^Ala mutation on one or both alleles of *APP* prevents all the memory and synaptic deficits found in the FDD_KI_ mice. This is seen in a short-term memory test, such as the RAWM task, and also in an ethologically relevant, non-aversive behavioral context, such as the NOR task. The memory deficits were prevented at their start and no deficits could be found even as late as 9–12 months of age. The same was true for the synaptic plasticity at 12 months old. The FDD_KI_ mice show strong synaptic defects in the Schaffer collateral pathway, however, FDD_KI_/*APP^TA/TA^* the mice showed no such deficits.

In this context, it is worth noting that mutation of another phosphorylated amino acid present in the APP intracellular region, namely Tyr^682^, results in a different (almost opposite) phenotype. This tyrosine is comprised in the intracellular ^682^YENPTY^687^ sequence of APP, a docking region for numerous APP-binding proteins that regulate processing and functions of APP [19], [30]–[35]. Phosphorylation of Tyr^682^ is consequential. Some proteins, such as Grb2 [36], Shc [37], [38], Grb7 and Crk [39] interact with APP only when Tyr^682^ is phosphorylated; others, like Fe65, Fe65L1 and Fe65L2 only when this tyrosine is not phosphorylated [40], suggesting that phosphorylation–dephosphorylation on Tyr^682^ modulates APP functions. To test the *in vivo* function of Tyr^682^ we have created mice with Tyr^682^ replaced by a Gly. This knock-in mutation alters the function of APP in memory formation, development/aging [41], [42] and changes APP processing, leading to a significant decrease in Aß levels [29]. Thus, while the Thr^668^Ala mutation on *APP*, which does not reduce Aß production, prevents memory deficits of FDD_KI_ mice, the Tyr^682^Gly mutation, which reduces Aß production, causes cognitive defects on its own [41]. These data show that the intracellular region of APP has a fundamental role in memory formation, a role that is not linked to Aß.

**Figure 4:**
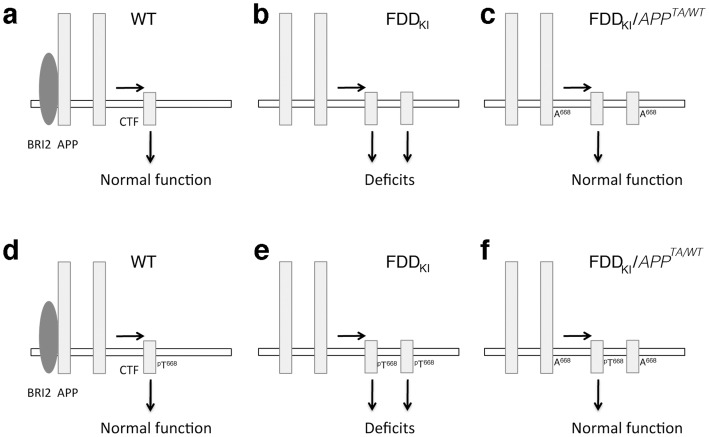
Model depicting the mechanisms by which Thr^668^ may lead to memory and synaptic plasticity deficits. (**a** and **b**), Due to loss of BRI2 protein, APP processing is increased during synaptic transmission and memory acquisition in FDD leading to increased production of ß-CTF. This event compromises synaptic plasticity and memory acquisition leading to memory deficits. (c), Thr^668^ is essential for the pathogenic role of ß-CTF, as shown by the evidence that mutating this residue into an Ala prevents development of memory/synaptic deficits. (d–f), Phosphorylation of Thr^668^ may be required or facilitate the synaptic-toxic role of ß-CTF, since the Thr^668^Ala mutation prevents phosphorylation.

New evidence points to β-derived metabolites of APP, especially ß-CTF, as the synaptic-toxic APP fragments mediating synaptic and memory impairments. The data presented here suggest that the synaptic-toxic activity of ß-CTF requires Thr^668^ (Figure 4a–c). It is possible that this synaptic-toxic activity necessitates or is enhanced by phosphorylation of Thr^668^ (Figure 4d–f), which is abolished by the Thr^668^Ala mutation. It is interesting to note that this mutation does not alter essential biological functions of APP during development [24], suggesting that targeting the role of Thr^668^, and perhaps its phosphorylation, in dementia may be an effective and safe therapeutic approach to dementias. Since the *APP^TA^* mutation prevents memory and synaptic deficits in heterozygosis, a partial reduction of the noxious pathogenic functions mediated by Thr^668^ will be therapeutically efficient.

## Methods

### Mouse Handling

The animals used for these studies were backcrossed to C57Bl6/J mice for at least 14 generations. Mice were handled according to the Ethical Guidelines for Treatment of Laboratory Animals of Albert Einstein College of Medicine. The procedures were described and approved in animal protocol number 200404. The Institutional Animal Care and Use Committee (IACUC) approved this protocol. IACUC is a federally mandated committee that oversees all aspects of the institution’s animal care and use program, facilities and procedures. The regulations of the USDA and PHS require institutions using animals to appoint an IACUC. The members of the IACUC are appointed by the Dean of Albert Einstein College of Medicine of Yeshiva University (Einstein).

### Synaptosomes Preparations

Hippocampi were homogenized in H buffer [5 mM Hepes/NaOH pH 7.4, 1 mM EDTA, 1 mM EGTA, 0.32 M sucrose, plus phosphatase/protease inhibitors at 10% (w/v) and centrifuged at 800 g for 10 min. The supernatant (S1) was separated to supernatant (S2) and pellet (P2) by spinning at 9,200 g for 15 min. P2 represents crude synaptosomal fraction.

### Antibodies

The following antibodies were used: anti-APP (Chemicon), anti-APP-CTF (Invitrogen), anti-APP^p^Thr^668^ and : anti-Akt (Cell Signaling). Secondary antibodies conjugated with horse-radish-peroxidase are from Southern Biotechnology.

### Electrophysiology and Behavior

Only male mice were used to avoid variations due to hormonal fluctuations during the estrous female cycle, which influence severely behavioral and electrophysiological tests.

### Spatial Working Memory

A six-armed maze was placed into white tank filled with water (24–25°C) and made opaque by the addition of nontoxic white paint. Spatial cues were presented on the walls of the testing room. At the end of one of the arms was positioned a clear 10 cm submerged platform that remained in the same location for every trial in 1 d but was moved approximately randomly from day to day. On each trial, the mouse started the task from a different randomly chosen arm. Each trial lasted 1 min, and errors were counted each time the mouse entered the wrong arm or needed more than 10 s to reach the platform. After each error, the mouse was pulled back to its starting position. After four consecutive acquisition trials, the mouse was placed in its home cage for 30 min, then returned to the maze and administered a fifth retention trial. The scores for each mouse on the last 3 days of testing were averaged and used for statistical analysis.

### Visible Platform Testing

Visible platform training to test visual and motor deficits was performed in the same pool as in the RAWM; however, the arms of the maze were removed. The platform was marked with a black flag and positioned randomly from trial to trial. Time to reach the platform and speed were recorded with a video tracking system (HVS 2020; HVS Image).

### Open Field and Novel Object Recognition

After 30 min to acclimate to the testing room, each mouse was placed into a 40 cm×40 cm open field chamber with 2 ft high opaque walls. Each mouse was allowed to habituate to the normal open field box for 10 min, and repeated again 24 hours later, in which the video tracking system (HVS 2020; HVS Image) quantifies the number of entries into and time spent in the center of the locomotor arena. Novel object recognition was performed as previously described [43]. Results were recorded as an object discrimination ratio (ODR), which is calculated by dividing the time the mice spent exploring the novel object, divided by the total amount of time exploring the two objects.

### Electrophysiology

Transverse hippocampal slices (400 µm) were transferred to a recording chamber where they were maintained at 29°C and perfused with artificial cerebrospinal fluid (ACSF) continuously bubbled with 95% O_2_ and 5% CO_2_. The ACSF composition in mM was: 124 NaCl, 4.4 KCl, 1 Na_2_HPO_4_, 25 NaHCO_3_, 2 CaCl_2_, 2 MgSO_4_, and 10 glucose. CA1 field-excitatory-post-synaptic potentials (fEPSPs) were recorded by placing both the stimulating and the recording electrodes in CA1 stratum radiatum. For LTP experiments, a 30 min baseline was recorded every minute at an intensity that evoked a response approximately 35% of the maximum evoked response. LTP was induced using a teta-burst stimulation (four pulses at 100 Hz, with bursts repeated at 5 Hz and each tetanus including one ten-burst train). Responses were recorded for 90 min after tetanization and plotted as percentage of baseline fEPSP slope.

### Statistical Analysis

All data are shown as mean ± s.e.m. Experiments were performed in blind. Statistical tests included two-way ANOVA for repeated measures and t-test when appropriate.
